# Optimizing the scale-up production of fermented astragalus and its benefits to the performance and egg quality of laying hens

**DOI:** 10.3389/fmicb.2023.1165644

**Published:** 2023-04-26

**Authors:** Weiwei Dong, Zhanlei Fan, Panxian Li, Jun Liu, Guoping Sun, Nan Peng, Yunxiang Liang, Shumiao Zhao

**Affiliations:** ^1^State Key Laboratory of Agricultural Microbiology and College of Life Science and Technology, Huazhong Agricultural University, Wuhan, China; ^2^Hubei Poder Biotechnology Co., Ltd., Huangshi, China

**Keywords:** fermented astragalus, scale-up production, egg quality, intestinal microbiota, laying hens

## Abstract

Astragalus is a homologous medicine and food that benefits human beings and poultry rearing. Fermented astragalus (FA) is a valuable product obtained by fermentation, but its scale-up production requires optimization and expansion of solid-state fermentation (SSF). In this study, *Lactobacillus pentosus* Stm was screened as the most suitable LAB strain for fermenting astragalus due to its excellent capacity. After optimization and expansion of SSF, LAB count and lactic acid content reached 206 × 10^8^ cfu/g and 15.0%, respectively. Meanwhile, the content of bioactive compounds in FA was significantly enhanced. Feeding experiments with laying hens indicated that supplementing FA in the diet significantly improved the performance and egg quality, as evidenced by reduced feed-to-egg ratio and egg cholesterol. This was due to the promotion of intestinal health by shifting intestinal microbiota. Therefore, this is a systematical endeavor of producing scaled-up FA with promising potential as a feed additive in the poultry breeding industry.

## Introduction

Astragalus (*Astragalus membranaceus*) is a perennial medicinal herb, whose root has been used as a traditional Chinese medicine for centuries, according to the *Compendium of Materia Medica*. This book noted various health benefits provided by the high medicinal value of astragalus, making it one of the top-favored homologous medicines and foods in China (Wang et al., 2023). Astragalus has also been widely applied as a supplement in the poultry farming industry to confer a variety benefits to hosts. Studies have shown that astragalus is good for fiber digestion, average daily gain, rumen fermentation, and antioxidant capacity in sheep (Wang et al., 2021a,b), as well as growth performance and immune response in the lamb (Hao et al., 2020), growth performance, and intestinal health in carp (Shi et al., 2022), and the alteration of fecal microbiota in laying hens (Qiao et al., 2018b). The bioactive compounds responsible for these benefits include polysaccharides, saponins, flavonoids, and many other trace components in astragalus (Li et al., 2009, 2014; Ibrahim et al., 2013; Auyeung et al., 2016; Shi et al., 2021; Zhou et al., 2021; Qiao et al., 2022). However, adding astragalus directly into feed can have side effects, such as anti-nutritional factors and a strong odor.

Fermentation is a powerful method for modifying traditional Chinese medicine, enhancing positive effects and alleviating side effects (Li et al., 2020; Son et al., 2020). To date, many strains combined with different fermentation methods have been used to process astragalus. *Streptococcus alactolyticus* FGM was used to increase the yield of polysaccharides during astragalus fermentation (Zhang et al., 2011), while solid-state fermentation of astragalus by *Aspergillus oryzae* M29 was found to boost the phenolic antioxidant activity (Sheih et al., 2011). Qiao et al. applied *Bacillus subtilis* to treat astragalus *via* liquid fermentation, and the production of polysaccharides reached as high as 4.93% (Qiao et al., 2017). Subsequently, lactic acid bacteria (LAB), consisting of *Lactobacillus plantarum* and *Enterococcus faecium*, were used to ferment astragalus and increase the production of polysaccharides, saponins, and flavonoids in fermented astragalus (FA) (Qiao et al., 2018c). When *L. plantarum*-FA was added to the diet of broilers, it improved their growth performance and serum biochemical parameters, as well as the performance and serum antioxidant ability of laying hens (Qiao et al., 2018a; Shi et al., 2020). Moreover, *B. subtilis*-FA exhibited therapeutic effects on hyperuricemia *via* modulation of gut microbiota in mice models (Wang et al., 2023). Fermentation is thus an effective method to increase the content of bioactive substances in astragalus, and FA could be utilized as a feed additive for poultries and even as a potential medicine for some illnesses to relieve symptoms.

Although previous studies have shown that fermentation elevates some bioactive substances and that FA exhibits positive effects on poultry and some illness models, few reports have studied the mechanism behind these positive effects, especially from the perspective of intestinal microbiota. Additionally, the elaborate fermentation processes of FA were not recorded. Hence, it is necessary to optimize the fermentation processes of FA and scale up to speed up practical application in the future. In this study, we optimized the fermentation processes from many factors, including starting strain, inoculation ratio, glucose addition, and temperature, to increase the LAB count, lactic acid content, and bioactive compounds in FA. Moreover, the fermentation scale was gradually expanded from 500 g to 1000 kg, achieving plant-scale production and promoting practical application. Meanwhile, the effectiveness of FA was evaluated by conducting feeding experiments on laying hens. Specifically, we used FA as a feed additive for laying hens and clarified their performance, egg quality, serum immunity, and intestinal microbiota. Finally, the mechanism behind the positive effects of FA on laying hens would be explained from the perspective of intestinal microbiota. Therefore, an efficient process was developed to produce FA at the plant scale, which could be applied as a beneficial feed additive to improve the intestinal health and egg quality of laying hens.

## Materials and methods

### Screening of LAB strains for FA production

The raw astragalus powder was obtained from Hubei Poder Biotechnology Co., Ltd, Huangshi, China, and its composition is presented in Supplementary Table S1. In total, 13 LAB strains, including *Lactobacillus paracasei, Lactobacillus casei, Lactobacillus pentosus, Pediococcus acidilactici*, and *Lactobacillus plantarum*, stored in our laboratory were evaluated for their ability to ferment astragalus using solid-state fermentation (SSF). Each LAB strain was separately inoculated into 500 g of the mixture (astragalus: water =1:1) and placed in sealed fermentation bags, followed by incubation at 37°C for 48 h. After fermentation, the LAB count and lactic acid content in FA were detected. For this purpose, 10 g of fresh FA was added into 90 ml of sterile ddH_2_O and shaken at 160 rpm for 20 min. Afterward, supernatants were diluted and plated on MRS agar to enumerate the number of LAB cells (Dong et al., 2022b). Simultaneously, 5 g of dried FA was added into 45 ml of sterile ddH_2_O, shaken at 220 rpm for 30 min, and then centrifuged at 12000 rpm for 5 min. The supernatant was filtered through a 0.22-μm filter for lactic acid measurement by high-performance liquid chromatography (HPLC) (Dong et al., 2022b).

### Optimization for FA production

Based on the screening results, *L. pentosus* Stm (CGMCC 1.2439) was selected as the starting LAB strain for SSF of astragalus due to its superior ability to proliferate and produce lactic acid. The basic SSF parameters were designed as follows: 250 g of astragalus was mixed with 250 g of water, and *L. pentosus* Stm was inoculated at a ratio of 3%. The mixture was then placed in a fermented bag and incubated at 37°C for 48 h. However, several factors, including the addition of bran, feed-water ratio, inoculation ratio of L. pentosus Stm, glucose addition, and enzyme addition, may affect the SSF of astragalus. Hence, optimization of SSF was performed based on the above factors at different levels (Supplementary Table S2), and an orthogonal test was conducted for enzyme addition (xylanase, cellulase, and pectinase) (Supplementary Table S3). The levels of each factor were tested separately with four duplicates and assessed based on the number of LAB cells and lactic acid content.

### Producing FA from the lab scale to the plant scale

After optimizing the parameters mentioned above, we determined the optimal condition for producing FA. The optimal condition was as follows: 25% bran addition, a feed–water ratio of 1:1.1, an initial inoculation ratio of *L. pentosus* Stm at 3%, glucose addition at 1%, xylanase addition at 0.3%, cellulase addition at 0.2%, and pectinase addition at 0.3%. We then scaled up the SSF process to 10 kg of each fermented bag and optimized the fermentation temperature (30°C, 33°C, 36°C, 39°C, and 42°C) and time (48 h, 60 h, 72 h, 84 h, 96 h, 108 h, and 120 h). The number of LAB cells and lactic acid content at each level were measured according to the above methods.

Finally, the plant-scale production at 1000 kg of SSF was carried out under an optimal condition in a relatively open environment. A five-point sampling method was applied to collect samples every 12 h for measuring the content of total flavonoids, saponins, and astragalus polysaccharides during SSF (Supplementary material method part). Because the fermentation was performed in a relatively open environment and the raw materials were not sterilized, it was essential to decode the microbial community and avoid contamination from pathogens. Thus, the microbial community of the above samples was analyzed via amplicon sequencing every 24 h. After completion of the FA product, it was dried at 45°C until the moisture content was lower than 10% and then stored for further feeding experiments.

### Supplementary of FA in the diet for laying hens

The feeding experiment was carried out at a laying hen breeding plant (Xiaogan, China). Here, three experimental groups (Table 1) were set to determine the effect of FA on the performance and health of laying hens. A total of 384 laying hens (Nonda-5, 42 weeks) were randomly assigned to one of the three groups, with four duplicates per group. Each replicate comprised 32 hens, and eight hens were housed in each cage to ensure adequate living space and feed availability. The CK group was fed a normal diet throughout the 28-day feeding cycle, with nutrient content based on the recommended values from the National Research Council (1994), as presented in Supplementary Table S4. The FA group received a diet that included 1% FA mixed with 99% normal feed, while the UA group received a diet that included 1% UA mixed with 99% normal feed. Before the start of the feeding experiment, all laying hens were given a week to adapt to the normal feed. Subsequently, the feed was changed according to the group setting, and the feeding regimen continued for 28 days. During the experiment, feeding was conducted four times per day (at 5:00, 10:00, 14:00, and 18:00), and hens had free access to fresh water. The room temperature was maintained at 25°C, and lighting was provided for 16 h per day (from 4:00 to 20:00). Eggs laid by the hens in each group were collected at 13:00 each day.

**Table 1:** Parameters of experiment groups for feeding laying hens.

**Group sets**	**Feeding parameters**	**Duplicates**	**Hens in each duplicate**
Control check (CK)	Normal feed	4	32
Fermented Astragalus (FA)	1% Fermented Astragalus + normal feed	4	32
Unfermented Astragalus (UA)	1% Unfermented Astragalus + normal feed	4	32

### Performance and egg quality

The number and weight of eggs from each group were recorded daily, and feed consumption was calculated weekly. Various performance indicators were evaluated, including total egg weight per day, feed-to-egg ratio, egg-laying rate, and the percentage of bad eggs. To assess the egg quality, four eggs from every duplicate of each group were randomly gathered weekly, thus collecting 96 eggs per group during the whole feeding phase. Here, the nutrient content of the egg, such as protein, lipid, and cholesterol, was detected according to national standards GB/T 5009.5-1985, GB/T 5009.6-2003, and GB/T 37077-2018, respectfully. Subsequently, the egg density was measured via the floating method with a saline solution. The egg shape index was calculated as the ratio of the length of the major axis to the length of the minor axis. The shell strength was detected via a special micrometer, while thick albumen, Haugh unit, and yolk color were determined by an egg analyzer (NABEL DET-6000, Tokyo, Japan).

### Immunity and intestinal microbiota

After completing the feeding experiments, the immunity and intestinal microbiota of birds were analyzed. Here, two hens from every duplicate of each group (eight hens for each group) were randomly chosen to collect blood, heart, liver, spleen, and cecal content. Blood samples were pretreated by centrifugation at 1200 *g* for 10 min to collect serum. The heart, liver, spleen, and body of hens were weighed to calculate the related indexes by dividing the organ weight by body weight. The collected serum and cecal content samples were immediately stored in dry ice for ease of transfer and later stored at −80°C for further study. The serum samples were then used to detect immunity indexes, including immunoglobulin A (IgA), immunoglobulin M (IgM), immunoglobulin Y (IgY), interleukin-2 (IL-2), interleukin-6 (IL-6), and tumor necrosis factor-α (TNF-α), via an ELISA kit (RENJIE bio, Shanghai, China). Moreover, the cecal content samples were used for DNA extraction and amplicon sequencing.

### Amplicon sequencing

Here, FA and cecal content samples were used for total DNA extraction, library construction, and high-throughput sequencing. A total of 0.2 g of sample was taken to extract the total DNA based on the instruction of the E.Z.N.A.^®^ DNA kit (Omega Bio-Tek, USA). After a quality check of DNA by agarose (0.8%) gel electrophoresis and the concentration detection via NanoDrop 2000 UV (Thermo, USA), 20 ng of extracted DNA in each sample was used as the template to construct the V3–V4 region of the 16S rRNA gene library. The primer pair of 338F (ACTCCTACGGGAGGCAGCA) and 806R (TCGGACTACHVGGGTWTCTAAT) was applied with Q5 high-fidelity DNA polymerase (NEB, USA) for PCR using the following parameter conditions: 98 for 5 min, 98°C for 30 s, 55°C for 30 s, and 72°C for 25 s for 28 cycles, and finally at 72°C for 5 min. After amplification, the purified 16S rRNA gene library was sequenced by the Illumina MiSeq platform.

### Data analysis and data availability

All data were processed by Excel (version 2019), and the difference was evaluated by one-way ANOVA with the software SPSS (version 24.0, SPSS Inc., Chicago, USA). Here, the significance at *p* < 0.05 was conducted under Duncan's multiple range test. The bioinformatics analysis of amplicon sequencing was carried out with QIIME 2 (2019.4) according to our previous study (Dong et al., 2022b). Raw sequences generated by sequencing in the present study have been deposited to NCBI with Bio project accession number PRJNA886379.

## Results

### Screening of the LAB strains for FA production

**Figure 1:**
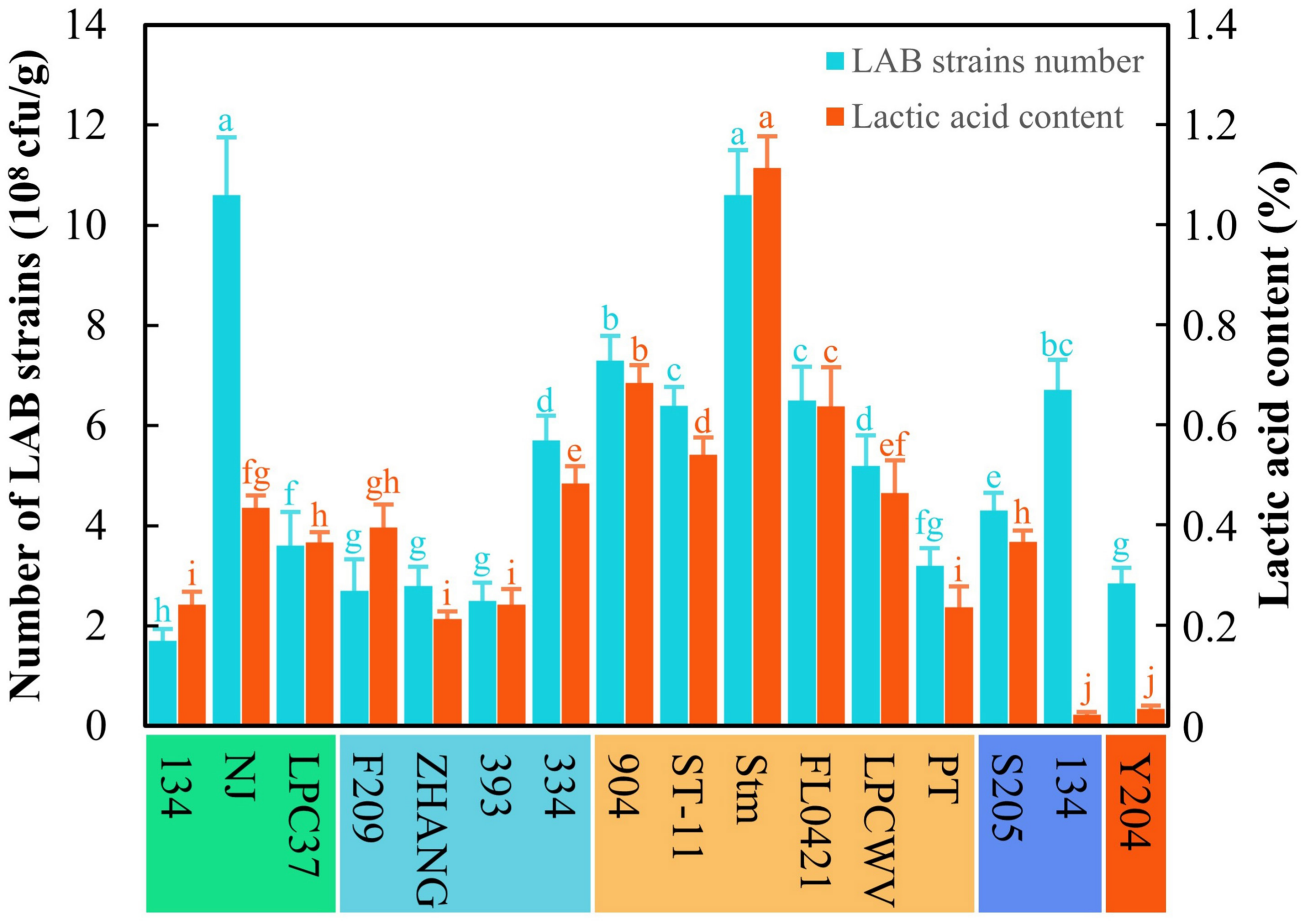
Screening of LAB strains for producing fermented astragalus. Here, five species of LAB were used, namely *L*. *paracasei* (134, NJ, LPC37), *L*. *casei* (F209, ZHANG, 393, 334), *L*. *pentosus* (904, ST-11, Stm, FL0421, LPCWV, PT), *P*. *acidilactici* (205, 134), and *L*. *plantarum* (Y204) (one-way ANOVA was performed to evaluate the difference). The same letter showed no significant difference between the chosen groups, while the different letters revealed that significant difference existed between the chosen groups.

The 13 LAB strains stored in our laboratory were tested for their abilities to ferment astragalus by SSF based on the strain count and lactic acid content (Figure 1). The results revealed that the strain *L. pentosus* Stm was the applicable one to ferment astragalus among all the LAB strain stored in our laboratory due to the highest strain count (10.6 × 10^8^ cfu/g) and lactic acid content (1.1%). Meanwhile, all LAB strains used astragalus as the substrate to grow and proliferate, thus achieving the strain count at a level of 10^8^ cfu/g. However, the lactic acid production among these LAB strains differed from each other even in orders of magnitude. Despite that, *L*. *paracasei* NJ gained the same strain count as *L. pentosus* Stm, but its lactic acid content only reached 0.4%, which was much lower than that of *L. pentosus* Stm. Hence, *L. pentosus* Stm was selected as the original strain for further production of FA.

### Optimizing and producing FA from the lab scale to the plant scale

**Figure 2:**
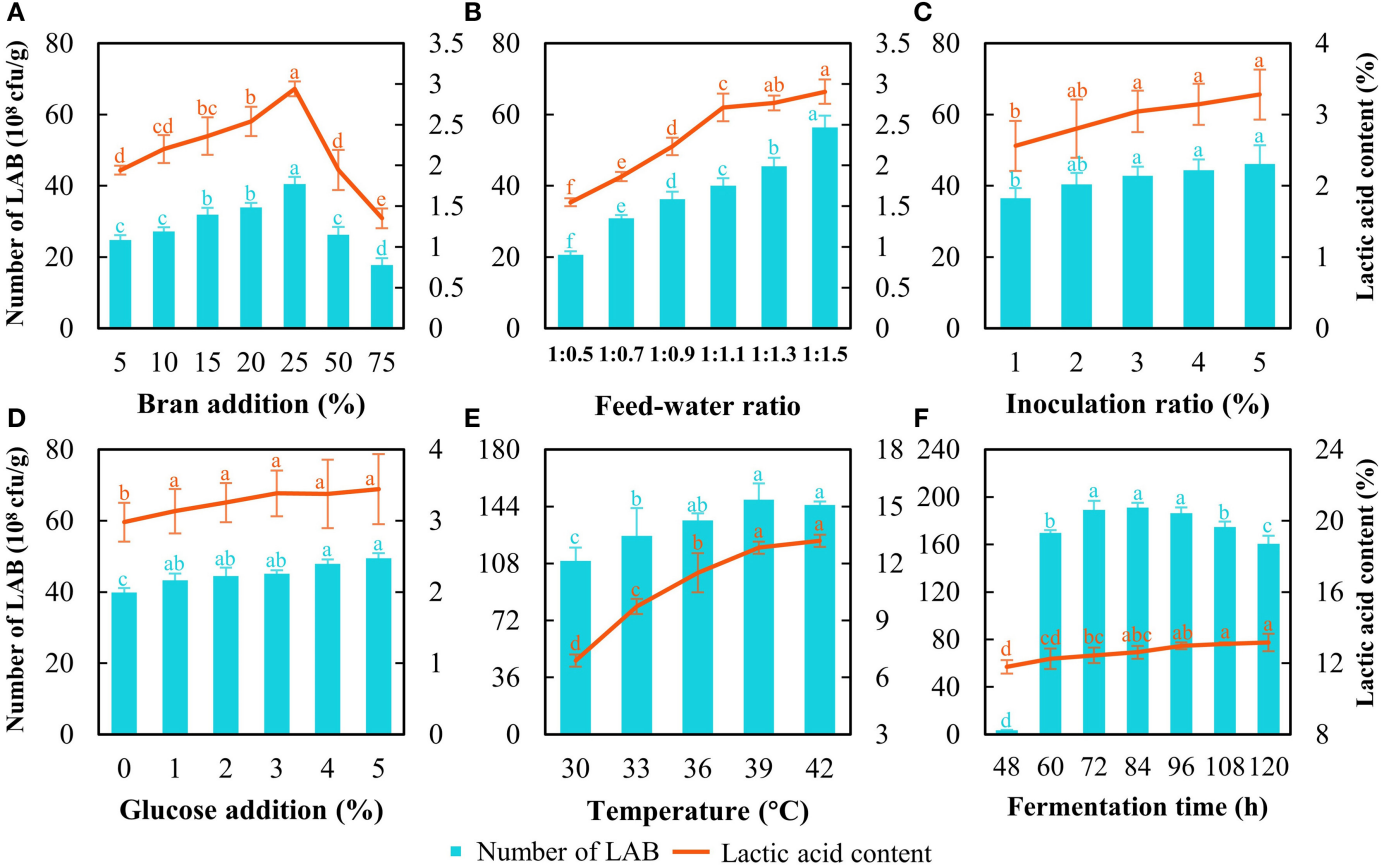
Optimization of SSF to produce fermented astragalus by detecting LAB count and lactic acid content. The factors include bran addition **(A)**, feed–water ratio **(B)**, inoculation ratio of *L. pentosus* Stm **(C)**, glucose addition **(D)**, fermentation temperature **(E)**, and fermentation time **(F**) (one-way ANOVA was performed to evaluate the difference). The same letter showed no significant difference between the chosen groups, while the different letters revealed that significant difference existed between the chosen groups.

First, the optimization of SSF at the lab scale was carried out initially with 250 g of astragalus. The results showed that the strain count and lactic acid content increased with the bran addition increasing from 5 to 25% and dropped when bran addition exceeded 25% (Figure 2A). The strain count and lactic acid content reached 40.5 × 10^8^ cfu/g and 3.0%, respectively, when the bran addition was at 25% (Figure 2A). Thereof, the bran addition was set at 25%. As for the feed–water ratio, the number of cells continued to increase with increasing water content, while the lactic acid content soared quickly and then this uptrend slowed down (Figure 2B). Although the increasing tendency of LAB count and lactic acid content was noted with the rise of water ratio during SSF, water accumulated and showed up at the bottom of fermented bags distinctly when the feed–water ratio went beyond 1:1.1. Considering the larger-scale production of FA at the relatively open environment and contamination risk from molds, the feed–water ratio was determined at 1:1.1. As for the inoculation ratio of *L. pentosus* Stm, the strain count and lactic acid content increased with increasing inoculation ratio; however, there was no significant difference in the value of those when the inoculation ratio exceeded 3% (Figure 2C). In this case, we chose inoculation of 3% as a base for further optimization. For glucose addition, there was no significant difference in lactic acid content and strain count when the addition was higher than 1% (Figure 2D). Higher addition of glucose represented a heavier cost, and the addition of glucose was decided at 1%.

Despite that a slight increase in strain count and lactic acid content being observed with the addition of enzymes, this rising tendency was negligible and not statistically significant when the addition exceeded 0.3% (Supplementary Figure S1). Then, the results of the orthogonal test on enzyme addition showed that the effects of the three factors on the lactic acid content followed xylanase > pectinase > cellulase according to the R-value (Table 2). These results indicated that the xylanase degrading hemicellulose in astragalus would yield xylose, which was a kind of fermentable sugar favored by *L. pentosus* Stm. Thus, the optimal addition for these three enzymes was with xylanase at 0.3%, cellulase at 0.2%, and pectinase at 0.3% (Table 2). Hence, the optimal SSF condition for producing FA was with bran addition at 25%, the feed–water ratio at 1:1.1, inoculation at 3%, and glucose addition at 1%, together with the above addition of enzymes. The strain count and lactic acid content reached 113.2 × 10^8^ cfu/g and 7.7%, respectively.

**Table 2:** Orthogonal test on enzyme addition for optimization of SSF.

**Number**	**A (xylanase, %)**	**B (cellulase, %)**	**C (pectinase, %)**	**Lactic acid content (%)**
1	0.1	0.1	0.1	5.86
2	0.1	0.2	0.3	6.34
3	0.1	0.3	0.2	5.74
4	0.2	0.1	0.3	5.26
5	0.2	0.2	0.2	5.08
6	0.2	0.3	0.1	4.92
7	0.3	0.1	0.2	6.48
8	0.3	0.2	0.3	7.70
9	0.3	0.3	0.1	7.62
K1	6.0	5.9	6.2	
K2	5.1	6.4	5.8	
K3	7.3	6.1	6.4	
R	2.18	0.50	0.64	
Optimal level	A3	B2	C3	
Factor	A>C>B			

The results of temperature optimization indicated that 39°C was appropriate since the LAB count was the highest together with the second highest lactic acid content (Figure 2E). Although a higher temperature (42°C) achieved higher lactic acid content than that at 39°C, more energy would be consumed during manufacturing in a factory. When considering the fermentation time, a typical growth curve of *L. pentosus* Stm was found (Figure 2F). The exponential phase ended at 72 h, the stationary phase continued from 72 h to 96 h, and then the death phase began. The LAB count at the stationary phase (72–96 h) was the highest with no difference. The lactic acid content increased during SSF, but no significance was found over 96 h. After entering the death phase, the LAB count decreased rapidly, and the cells of *L. pentosus* Stm would compete with each other by degrading the bioactive substances in FA and even producing some unbeneficial substances (Rezaei, 2022). Meanwhile, longer fermentation time would prolong the running time of the machine and lead to higher costs when produced on a large scale. Hence, we chose 96 h as the fermentation time for further production.

### Bioactive compounds and bacterial community in FA

**Figure 3:**
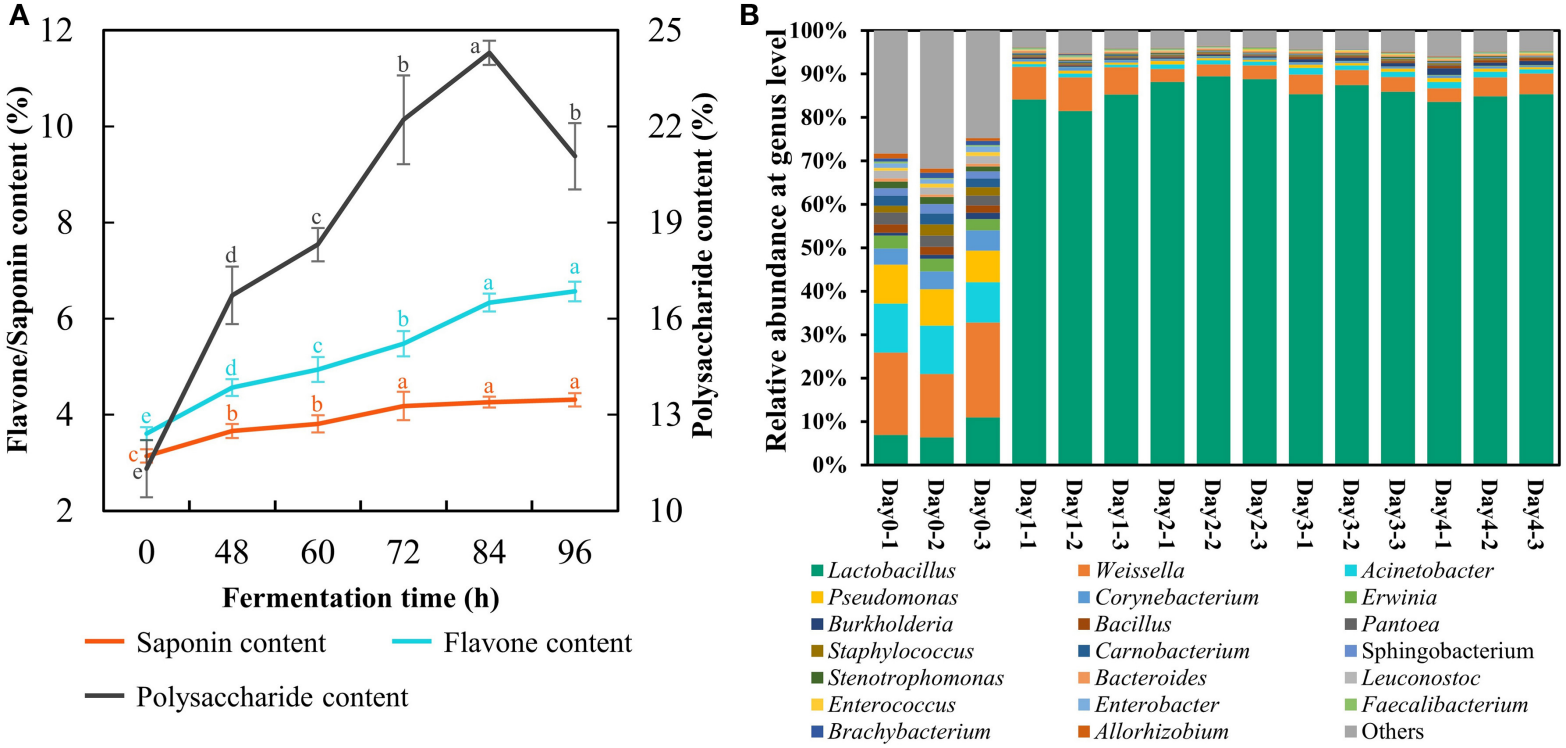
Changes of bioactive compounds **(A)** and bacterial community **(B)** in fermented astragalus at plant scale (one-way ANOVA was performed to evaluate the difference). The same letter showed no significant difference between the chosen groups, while the different letters revealed that significant difference existed between the chosen groups.

Here, the FA was produced at the plant scale with 1 ton of materials fermented at 39°C persisting for 96 h under the above optimal parameters. The strain count and lactic acid content reached 206 × 10^8^ cfu/g and 15.0%, respectively. The total flavonoids and saponins presented an uptrend during SSF terminating at a content of 6.6% and 4.3% respectfully (Figure 3A). The astragalus polysaccharides reached the peak at 84 h with a value of 24.3% but descended at 96 h (Figure 3A), indicating that fermentation time should not be too long because *L. pentosus* Stm would consume astragalus polysaccharides for survival when entering the death phase. Furthermore, the duration of SSF with 96 h was suitable for ensuring the high concentration of these bioactive substances to manifest benefits.

The bacterial community of FA was detected to investigate whether there were pathogens in the final fermented product (Figure 3B). The results suggested that potential (opportunistic) pathogens including *Acinetobacter, Erwinia, Burkholderia, Pantoea*, and *Staphylococcus* accounted for 35.5% in raw material. The abundance of these potential pathogens significantly decreased after SSF, and their final proportion was lower than 4.5%. Meanwhile, *Lactobacillus* became the most dominant bacteria after SSF with its abundance reaching 84.6% on day 4, which was consistent with the results of Qiao's study that the prevalent genus in FA by *L. plantarum* was *Lactobacillus* on day 3 (Qiao et al., 2018c). However, some potential (opportunistic) pathogens recaptured the ecological niche within the SSF lasting for 30 days (Qiao et al., 2018c). From these results, we could conclude that SSF with *L. pentosus* Stm persisting for 4 days evidently inhibited the growth of potential pathogens together with enriching bioactive substances and *Lactobacillus*, a generally safe LAB.

### The performance and egg quality of laying hens

**Figure 4:**
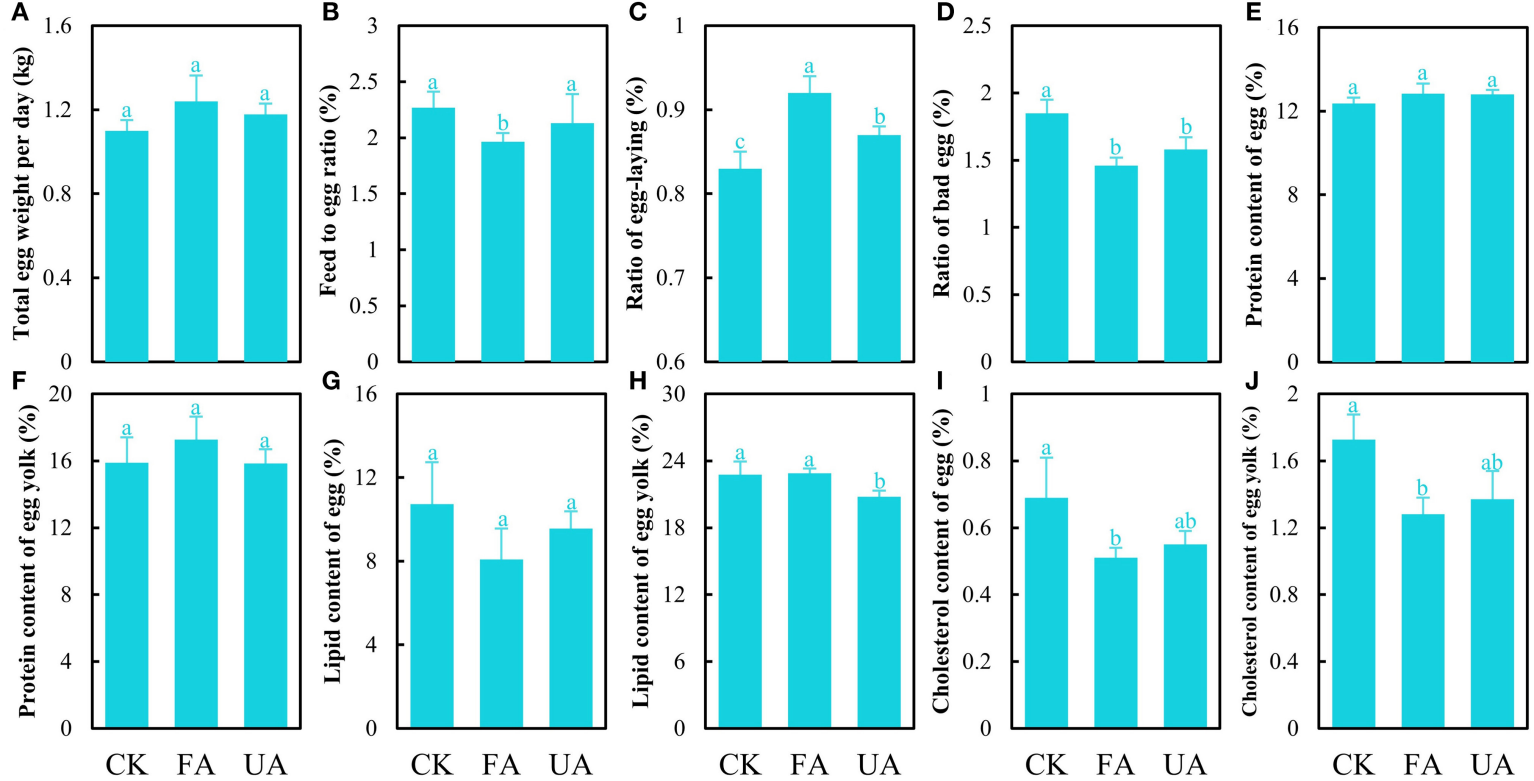
Performance and egg quality of laying hens after feeding experiments, including total egg weight per day **(A)**, feed-to-egg ratio **(B)**, ratio of egg-laying **(C)**, the ratio of bad egg **(D)**, protein of egg **(E)**, protein of egg yolk **(F)**, lipid of egg **(G)**, lipid of egg yolk **(H)**, cholesterol of egg **(I)**, and cholesterol of egg yolk **(J)** (one-way ANOVA was performed to evaluate the difference). The same letter showed no significant difference between the chosen groups, while the different letters revealed that significant difference existed between the chosen groups.

The effects of FA on the performance and egg quality of laying hens were investigated. The results showed that supplying FA in the diet exerted positive effects on the performance of laying hens (Figures 4A–D), and only the increase in total egg weight per day of the FA group was of no significance (Figure 4A). Importantly, adding FA in the diet significantly reduced the feed-to-egg ratio compared to the CK and UA groups (Figure 4B), which was similar to previous results (Shi et al., 2020), revealing that more masses of eggs were laid by feeding less diet *via* supplements of FA. Simultaneously, replenishing FA in the diet significantly accelerated the frequency of laying and reduced the ratio of bad eggs compared to the CK and UA groups (Figures 4C, D), indicating that more eggs were laid by the FA group but the average mass of laid eggs in the FA groups was not decreased (Supplementary Table S5). These results indicated that supplementing FA in diet boosted the performance of laying hens. Moreover, the quality of eggs from the FA group was evaluated as double A, which is higher than the CK and UA groups, based on the basic parameters of eggs (Supplementary Table S5). When considering the nutrient level of eggs, feeding FA exhibited no significant influence on the protein and lipid content of the egg quality (Figures 4E–H) but significantly reduced the cholesterol content of whole egg and egg yolk (Figures 4I, J). This advantage of the low cholesterol content in the eggs would be great for marketing and consumers, which could be developed into an egg product that highlighted low cholesterol.

### Health and immunity of laying hens

**Figure 5:**
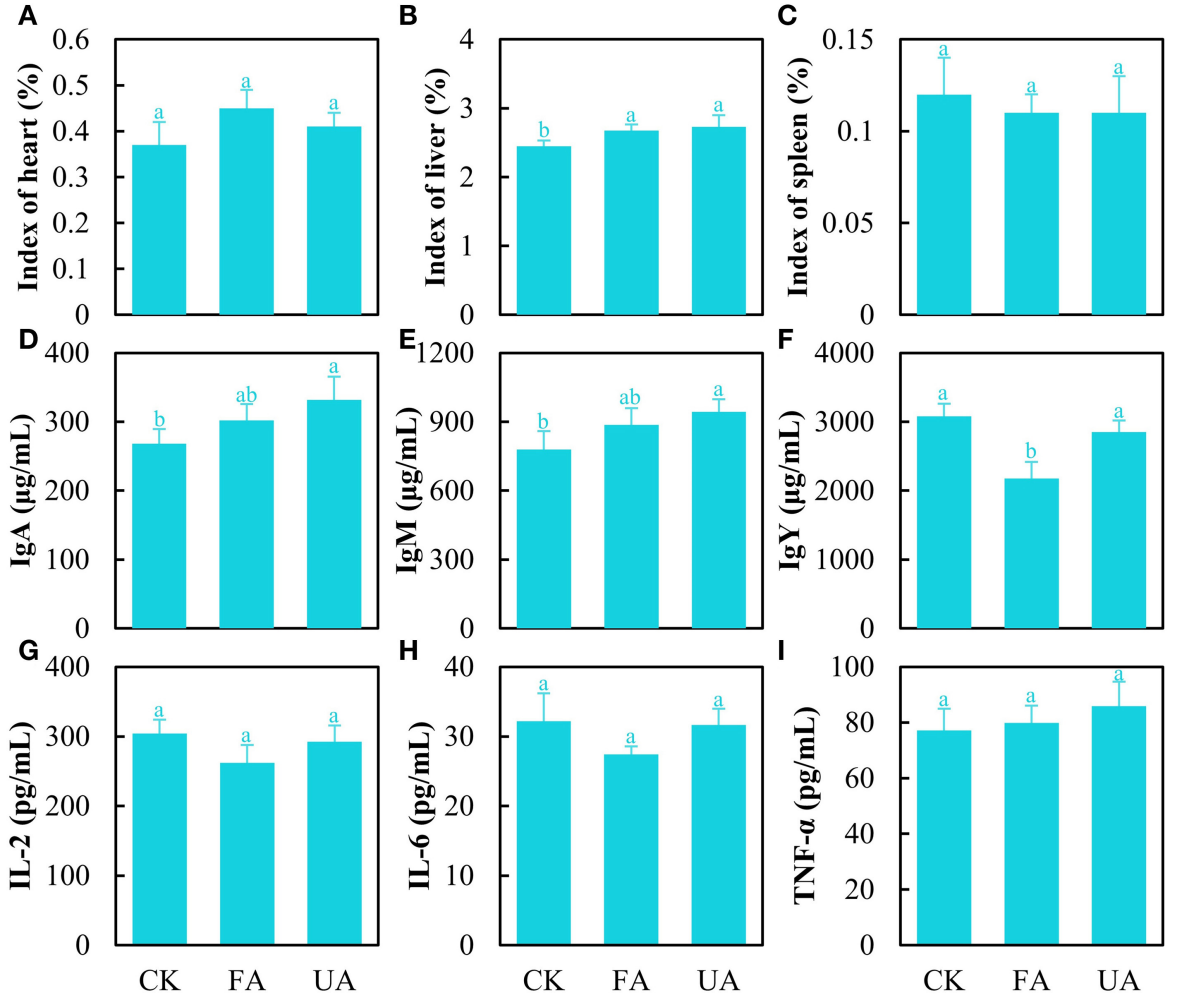
Immunity of laying hens after feeding experiments, including heart index **(A)**, liver index **(B)**, spleen index **(C)**, content of IgA **(D)**, content of IgM **(E)**, content of IgY **(F)**, content of IL-2 **(G)**, content of IL-6 **(H)**, and content of TNF-α **(I)** (one-way ANOVA was performed to evaluate the difference). The same letter showed no significant difference between the chosen groups, while the different letters revealed that significant difference existed between the chosen groups.

The organ indexes were analyzed encompassing the heart, liver, and spleen to estimate the health of laying hens (Figures 5A–C). The results stated that FA imparted no significant effect on heart and spleen indexes (Figures 5A–C). Interestingly, the liver index was significantly promoted in the FA and UA groups compared with the CK group (Figure 5B). As for the immunity of laying hens, several immune factors in serum were detected (Figures 5D–I). The level of immune factors manifested the ability of the body to respond to pathogens and viruses (Chaplin, 2010). As for IgA and IgM, the concentrations of the UA group were significantly higher than those of the CK group (Figures 5D, E). The pathogens in UA (day 0 in Figure 3B) caused these high levels of IgA and IgM, while fermentation reduced the abundance of pathogens thus bettering this. Similarly, the concentrations of IL-2 and IL-6 in the FA group were lower (no significance) compared with the CK and UA groups (Figures 5G, H), suggesting that fermentation may have reduced the virus content, as IL-2 and IL-6 levels are associated with viral infections. Unexpectedly, the IgY concentration in the FA group was significantly lower than that in the CK and PA groups (Figure 5F), and the reason was unknown here. In addition, no difference was seen in the content of TNF-α among these three groups (Figure 5I).

### Intestinal microbiota of laying hens

**Figure 6:**
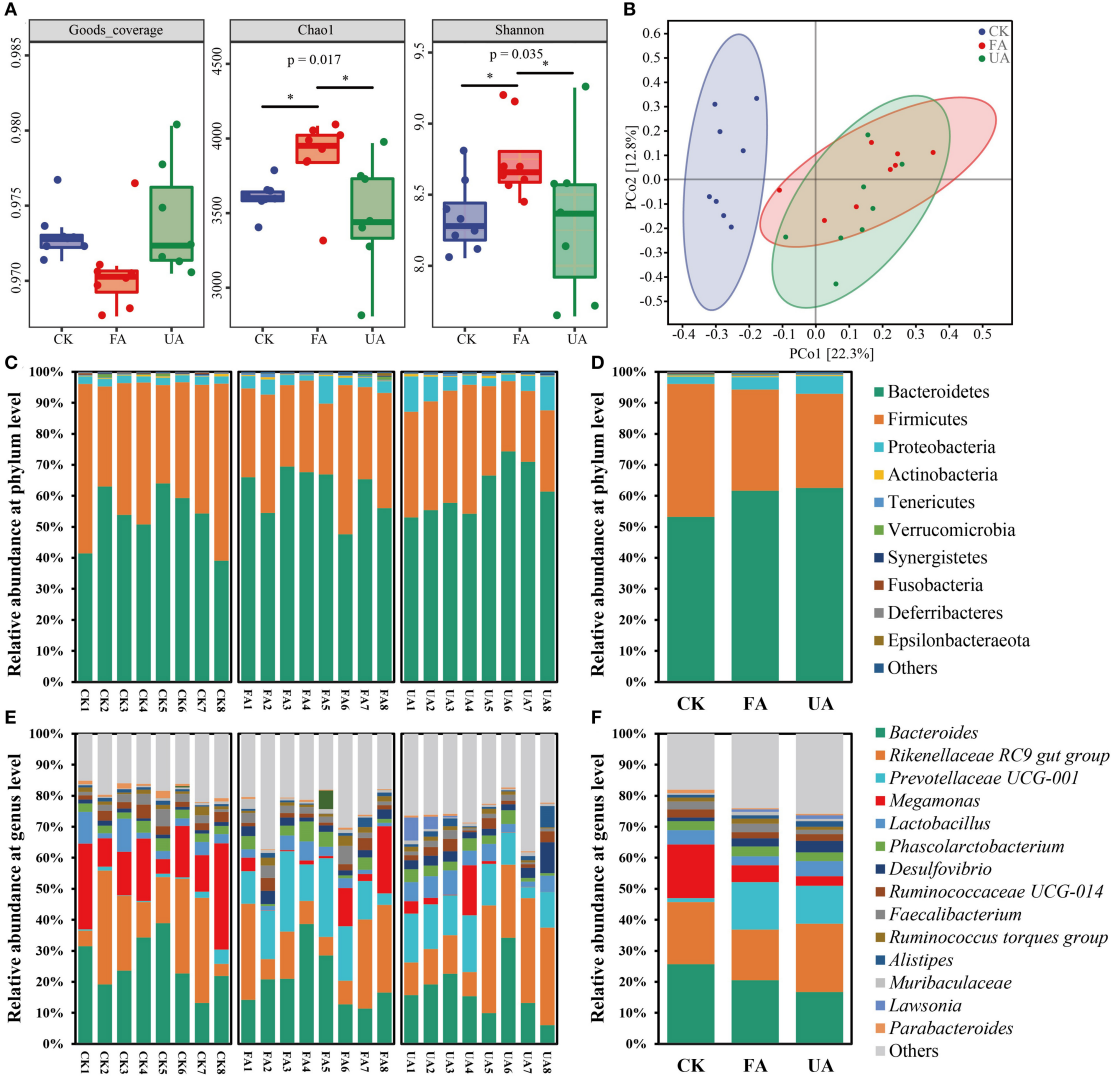
Intestinal microbiota of laying hens after feeding experiments, including α-diversity **(A)**, β-diversity **(B)**, bacterial composition at the phylum level **(C)**, bacterial composition at the phylum level on average **(D)**, bacterial composition at the genus level **(E)**, and bacterial composition at genus level on average **(F)**. Here, the Bray–Curtis distance was used with an authority of 0.95 to calculate the β-diversity.

In this study, the α-diversity, β-diversity, and composition of the microbial community were revealed (Figure 6). First, the value of goods coverage was all higher than 0.968 (Figure 6A), elucidating that the data from amplicon sequencing were of great depth and authenticity. The Chao1 richness of FA was significantly higher than that of the CK and UA groups (Figure 6A), revealing that more species in the FA group were found. As for the Shannon index, the value of the FA group was significantly higher than that of the CK and UA groups (Figure 6A). Thus, adding FA to feed increased the species diversity and evenness. Subsequently, the principal co-ordinates analysis (PCoA; Bray–Curtis distance with authority at 0.95) was used to evaluate the β-diversity of intestinal microbiota (Figure 6B). The results showed that supplement with FA or UA in feed significantly changed the β-diversity of intestinal microbiota (ANOSIM test, *R* = 0.3137, *p* = 0.001). Despite that, the symbols of the FA group were located closer to the CK group than those of the UA group at the PCo1 axis, which suggested that the FA group was more similar to the CK group compared with the UA group at β-diversity.

Although a supplement with FA in diet remarkably affected the diversity of intestinal microbiota in laying hens, the specific microbes that respond to these effects remain unknown. At the phylum level, Bacteroidetes, Firmicutes, and Proteobacteria were the dominant phyla (relative abundance > 1%) in these three groups (Figures 6C, D), which was in line with previous studies (Khan et al., 2020; Dai et al., 2022). Feeding with FA or UA increased the abundance of Bacteroidetes and Proteobacteria but decreased the abundance of Firmicutes (Figures 6C, D). At the genus level, 14 dominant genera were observed here, and *Bacteroides, Rikenellaceae RC9 gut group, Prevotellaceae UCG-001, Megamonas*, and *Lactobacillus* were the top five predominant genera (Figures 6E, F). Interestingly, the *Megamonas* was rarely reported in intestinal microbial of laying hens. Adding FA in feed elevated the abundance of *Prevotellaceae UCG-001* but decreased that of *Bacteroides, Rikenellaceae RC9 gut group, Megamonas*, and *Lactobacillus* compared with the CK group. Feeding with UA mainly increased the abundance of *Rikenellaceae RC9 gut group, Lactobacillus*, and *Desulfovibrio*; however, the abundance of *Bacteroides* and *Megamonas* was inhibited. Thus, supplying the diet with FA significantly altered the intestinal microbiota of laying hens in diversity and composition.

## Discussion

In summary, *L. pentosus* Stm was selected for fermenting astragalus among the tested LAB. Subsequently, the optimization based on the scaled-up production of FA was conducted through the single-factor optimization and orthogonal test. Accordingly, the plant-scale production of FA was achieved with LAB count and lactic acid content reaching 206 × 10^8^ cfu/g and 15.0%, respectively. Furthermore, the FA was applied as an additive in the diet for laying hens, and the laying performance and egg quality were promoted. In addition to these, the shift of intestinal microbiota by supplying FA in diet might account for the above positive effects on laying hens.

Here, *L. pentosus* species was capable of fermenting astragalus due to better lactic acid-producing ability and higher LAB count. The metabolic aptitude of hemicellulosic sugars and xylose in *L. pentosus* gained an advantage over other LAB species (Portilla-Rivera et al., 2010). Moreover, there are lots of xylose and partial hemicellulose in astragalus (Kallon et al., 2013), enhancing the fitness of substrates with *L. pentosus* when fermenting astragalus. Hence, *L. pentosus* stm was the suitable strain for further optimization of producing FA because of the highest LAB count and lactic acid content among these LAB.

When it came to the optimization of producing FA, the scale was gradually expanded from 500 g to 1 ton to satisfy the plant-scale production for future practical application. First, the single-factor optimization was carried out. Here, the bran provided extra reducing sugar (~4%) and crude protein (~19%) (Stevenson et al., 2012; Zhao et al., 2017) for the growth and metabolism of *L. pentosus* Stm, thus facilitating the LAB count and lactic acid content. The feed–water ratio impaired water content in SSF, which influenced the metabolic activity and rate of LAB. However, additional water will increase the water content of the final FA product, which is not conducive to the dry and storage (shelf-life) of products thus adding the cost (Dong et al., 2022a). As for the inoculation ratio and glucose addition, the turning point was chosen based on the consideration that further increases beyond this point would not yield significant gains but would result in heavier cost burdens. Subsequently, three enzymes were used to degrade the crude fiber (26%) in astragalus powder (Davis, 1973), providing extra fermentable sugars for the propagation and metabolism of *L. pentosus* Stm. Hence, the lactic acid content after optimization reached 7.7%, which was much higher than that (1.9%) of FA by *L. plantarum* (Qiao et al., 2018c). Afterward, the fermentation temperature and time were optimized. Here, fermentation temperature is associated with energy consumption, and fermentation time decided the production cycle, which influences the cost and running efficiency when manufacturing at a factory. After optimization of all these parameters, the LAB count and lactic acid content reached 186.5 × 10^8^ cfu/g and 13.0%, respectively, which increased 16-fold and 10-fold more than that before, indicating that optimization was effective and necessary.

After optimizing the production procedure of FA, the fermentation scale was expanded to 1 ton. The strain count and lactic acid content reached 206 × 10^8^ cfu/g and 15.0%, respectively, and this lactic acid content was about 50-fold than that of co-fermentation by *L. plantarum* and *Enterococcus faecium* (Qiao et al., 2018c). This increase might be caused by the different strains, mediums, and conditions used for fermentation. Moreover, the content of flavonoids, saponins, and polysaccharides significantly increased after SSF (Figure 3A). Flavonoids, saponins, and polysaccharides are the three main bioactive compounds in astragalus, which entails anti-inflammatory, immunoregulatory, anti-tumor, and anti-oxidative activities (Li et al., 2014; Auyeung et al., 2016). Meanwhile, SSF with *L. pentosus* Stm significantly enriched LAB and prohibited the potential pathogens (Figure 3B), escorting for the safety of the FA product. In addition, the lactic acid produced from SSF would improve the flavor and thus neutralize the smell of Chinese medicine, which improved the palatability of feed (Czech et al., 2009). Thus, the bioactive substances exhibiting benefits to hosts (Li et al., 2014; Auyeung et al., 2016; Ji et al., 2020), together with the probiotic capacity of *L. pentosus* (Izquierdo et al., 2017), made the FA a beneficial and safe additive for poultry.

Feeding experiments on laying hens revealed that a diet with FA increased the performance and egg quality by reducing the feed-to-egg ratio and cholesterol content of the egg, as well as facilitating the ratio of egg-laying. These positive effects might be attributed to the enhancement of the health status of the liver and the alteration of cholesterol metabolism in laying hens by supplying FA. Interestingly, lots of lipids were observed to surround and wrap the liver of laying hens in the CK group, but this phenomenon was not found in the FA group (figure not shown), outlining that FA could inhibit lipid synthesis, thus improving the liver's health status with higher liver index (Figure 5B). Moreover, the lipid synthesis was closely related to the cholesterol content in eggs (Iskender et al., 2017; Sarvestani et al., 2020). Here, cholesterol in an egg is synthesized *via* the liver and then transported by very-low-density lipoprotein (VLDL) (Dilawar et al., 2021). In addition, the flavonoids, saponins, and polysaccharides in astragalus were reported to improve the health status of the liver (Liu et al., 2014; Zhou et al., 2021). Hence, we speculated that the bioactive compounds in FA improve the health status of the liver and decreased the synthesis of lipids and VLDL, lowering the cholesterol of the egg, which needs further investigation.

**Figure 7:**
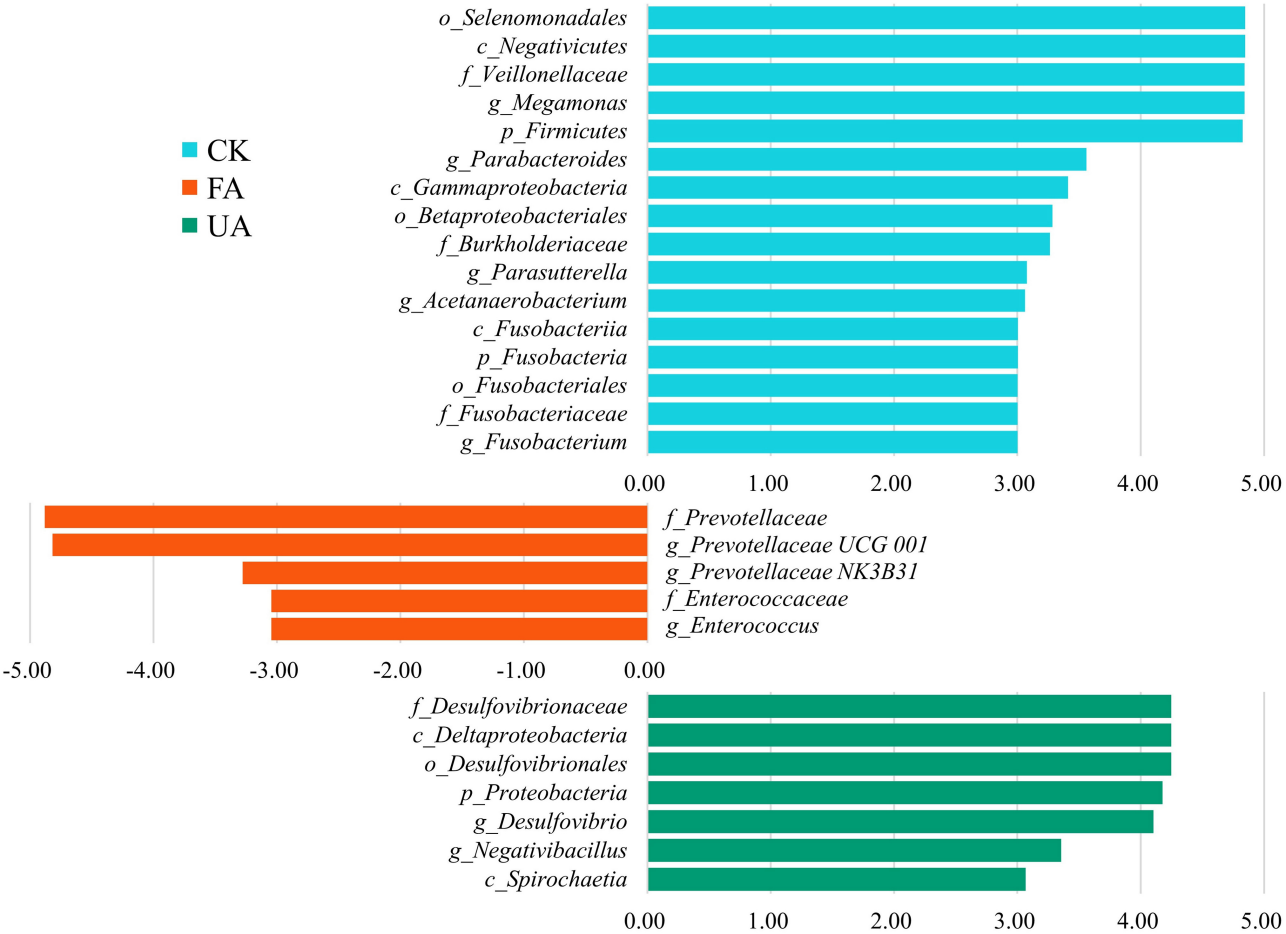
LEfSe analyses between each feeding group and CK group. Here, the LDA threshold was 3, and the Wilcoxon test was used.

Intestinal microbiota was associated with intestinal health, affecting the nutrition absorption and health of the host (Yeoman et al., 2012; Khan et al., 2020; Dai et al., 2022). The results revealed that supplements with FA in diet remarkably affected the diversity and composition of intestinal microbiota (Figure 6). At the phylum level, the rising abundance of Proteobacteria might account for the increase in the above liver index (Figure 5B), as high levels of Proteobacteria have been associated with liver development (Dai et al., 2022). Moreover, previous studies have found that the ratio of Firmicutes to Bacteroidetes is significantly associated with the host's ability to harvest energy (Kasai et al., 2015; Magne et al., 2020). In our study, the ratio of Bacteroidetes to Firmicutes in the FA and UA groups was lower than that in the CK group (0.55 and 0.52 vs. 0.86, respectively), which may have improved nutrient absorption and energy intake, leading to enhanced liver development in the FA and UA groups. To identify the specific microbes responsible for these positive effects at the genus level, we conducted an LDA effect size (LEfSe) analysis. *Megamonas, Parabacteroides, Parasutterella, Acetanaerobacterium*, and *Fusobacterium* were the core bacteria in the CK group (Figure 7). Here, *Parabacteroides* could improve host metabolism (Khan et al., 2020), while *Parasutterella* was involved in bile acid maintenance and cholesterol metabolism (Ju et al., 2019). *Acetanaerobacterium* was a common genus of chicken, and its function was currently unknown (Oakley et al., 2014). In the FA group, *Prevotellaceae UCG-001, Prevotellaceae NK3B31*, and *Enterococcus* were enriched as core bacteria, while *Desulfovibrio* and *Negativibacillus* became core bacteria in the UA group (Figure 7). The *Prevotellaceae UCG-001* and *Prevotellaceae NK3B31* could produce short-chain fatty acids, which benefit host health (Huang et al., 2021; Peng et al., 2022). *Enterococcus* could reduce serum cholesterol levels in chickens and improve egg quality (Abdel-Wareth, 2016), potentially leading to low cholesterol content in eggs. However, *Desulfovibrio* showed a significantly positive correlation with H_2_S production, generating adverse effects on intestinal health (Huang et al., 2019). *Negativibacillus* is a kind of pathogen in the intestinal tract, associated with gut dysbiosis (Tang et al., 2021). Therefore, FA promoted intestinal health by enriching *Prevotellaceae UCG-001, Prevotellaceae NK3B31*, and *Enterococcus*, while UA might gain adverse effects on intestinal health by increasing *Desulfovibrio* and *Negativibacillus*. These alterations in the intestinal microbiota by supplying FA benefited the intestinal health and hence enhanced the performance and egg quality of laying hens.

## Conclusion

In this study, we optimized the production of FA and expanded the scale from the lab scale to the plant scale. *L. pentosus* Stm was the applicable LAB to ferment astragalus due to its high LAB count and lactic acid content. After fermentation optimization and scale-up to 1 ton at the plant scale, the LAB count and lactic acid content of FA reached 206 × 10^8^ cfu/g and 15.0%, respectively, achieving increases over 18-fold and 12-fold, respectively. Meanwhile, the content of bioactive compounds in FA was significantly enhanced. Feeding experiments demonstrated that FA significantly facilitated the performance and egg quality by reducing the feed-to-egg ratio, decreasing the cholesterol content of the egg, and increasing the ratio of egg-laying. Dietary FA administration promoted intestinal health by enriching *Prevotellaceae UCG-001, Prevotellaceae NK3B31*, and *Enterococcus*, which were responsible for these beneficial effects. Therefore, our systematic optimization of FA production has promising application prospects as a feed additive for the poultry farming industry.

## Data availability statement

The datasets presented in this study can be found in online repositories. The names of the repository/repositories and accession number(s) can be found in the article/Supplementary material.

## Ethics statement

The animal study was reviewed and approved by Animal Care and Use Committee of Huazhong Agricultural University (HZAUCH-2019-008).

## Author contributions

WD: sampling, partial experiment, data processing, formal analysis, software, visualization, writing–original draft, and writing–review and editing. ZF: investigation, sampling, experiment, and data processing. PL: partial experiment and data processing. JL: sampling and resources. GS and YL: resources. NP: language edit. SZ: project administration, supervision, language editing, conceptualization, and funding acquisition. All authors contributed to the article and approved the submitted version.
