# Supplementary material for: Optimizing the scale-up production of fermented astragalus and its benefits to the performance and egg quality of laying hens
Source: Front Microbiol. 2023 Apr 26;14:1165644. doi: 10.3389/fmicb.2023.1165644 (PMC10169715; doi:10.3389/fmicb.2023.1165644)
Supplement: Supplementary file 1 [file Data_Sheet_1.docx]

***Frontiers in Microbiology***

**Optimizing the scale-up production of fermented astragalus and its benefits to the performance and egg quality of laying hens**

Weiwei Dong^1^, Zhanlei Fan^1^, Panxian Li^1^, Jun Liu^2^, Guoping Sun^2^, Nan Peng^1^, Yunxiang Liang^1^, Shumiao Zhao^1^*

^1^ State Key Laboratory of Agricultural Microbiology and College of Life Science and Technology, Huazhong Agricultural University, Wuhan (430070), China.

^2^ Hubei Boda Biology Co., LTD, Huangshi (435000), China.

*Corresponding authors:

Shumiao Zhao, Tel. /fax: +8602787281040; E-mail address: shumiaozhao@mail.hzau.edu.cn

Methods

The **total flavonoids content** in sample was detected based on Esmaeili’s study with slight revision (Khorasani Esmaeili et al., 2015). Firstly, the total flavonoids were extracted from 20 g of samples with 200 mL of 95% ethanol by heating reflux (twice). Then, 10 mL of extract was mixed with 1 mL of 5% NaNO_2_ and 1 mL of 10% Al(NO_2_)_3_, followed by 5 min incubation and the addition of 10 mL of 4% NaOH. The 95% ethanol was added to the constant volume of 25 mL, and absorbance at 500 nm was detected. At last, the total flavonoids content was determined according to the standard curve of rutin.

The **total** **saponins content** in sample was detected based on Le’s study with modification (Le et al., 2018). Firstly, the total saponins were extracted from 40 g of samples with 200 mL of 80% ethanol by heating reflux (twice), then adding 80% of ethanol to the constant volume of 250 mL, followed by incubation at room temperature overnight. Subsequently, 0.6 mL of extract was mixed with 0.5 mL of 8% vanillin and 5 mL of 72% H_2_SO_4_. After incubation at 62 °C for 20 min and cooling, absorbance at 538 nm was detected. At last, the total saponins content was evaluated according to the standard curve of astragaloside IV.

The **astragalus polysaccharides content** in sample was detected based on phenol sulfuric acid assay (Peng et al., 2022). Here, 1 g of sample was mixed with phenol solution (6%) and sulfuric acid (3 M). Then, the mixture was well shaken and placed in a boiling water bath for 30 min. Finally, absorbance at 490 nm was detected, and astragalus polysaccharides content was clarified according to the standard curve of glucose.

References:

1. Khorasani Esmaeili A, Mat Taha R, Mohajer S, Banisalam B. Antioxidant Activity and Total Phenolic and Flavonoid Content of Various Solvent Extracts from In Vivo and In Vitro Grown Trifolium pratense L. (Red Clover). Biomed Res Int. 2015:643285.
2. Peng Y, Ma F, Hu L, Deng Y, He W, Tang B. Strontium based Astragalus polysaccharides promote osteoblasts differentiation and mineralization. Int J Biol Macromol. 2022;205:761-771.
3. Le A, Parks S, Nguyen M, Roach P. Improving the Vanillin-Sulphuric Acid Method for Quantifying Total Saponins. Technologies. 2018;6(3):84.

Table S1 The basic composition of astragalus powder.

| Composition | Contents (%) |
| --- | --- |
| Moisture | 11.75 ± 0.18 |
| Carbohydrate | 49.67 ± 0.87 |
| Crude protein | 14.36 ± 0.62 |
| Crude fat | 4.55 ± 0.06 |
| Crude fiber | 3.82 ± 0.08 |
| Ash | 4.65 ± 0.11 |
| Polysaccharide | 11.20 ± 0.23 |
| Flavone | 3.12 ± 0.05 |
| Saponin | 3.60 ± 0.04 |

Table S2 Factors and levels of producing fermented astragalus during optimization.

| Factors | Levels |
| --- | --- |
| Bran addition | 5%, 10%, 15%, 20%, 25%, 50%, and 75% |
| Feed-water ratio | 1:0.5, 1:0.7, 1:0.9, 1:1.1, 1:1.3, and 1:1.5 |
| Inoculation ratio | 1%, 2%, 3%, 4%, and 5% |
| Glucose addition | 1%, 2%, 3%, 4%, and 5% |
| Xylanase addition | 0.1%, 0.2%, 0.3%, 0.4%, and 0.5% |
| Cellulase addition | 0.1%, 0.2%, 0.3%, 0.4%, and 0.5% |
| Pectinase addition | 0.1%, 0.2%, 0.3%, 0.4%, and 0.5% |

Table S3 Factors and levels of enzyme addition in orthogonal test during SSF.

| Factors  Levels | Xylanase addition | Cellulase addition | Pectinase addition |
| --- | --- | --- | --- |
| 1 | 0.10% | 0.10% | 0.10% |
| 2 | 0.20% | 0.20% | 0.20% |
| 3 | 0.30% | 0.30% | 0.30% |

Table S4 The composition of normal feed.

| Composition | Content (%) |
| --- | --- |
| Corn | 57 |
| Bean pulp | 24 |
| Sichuan pepper powder | 6 |
| Rock flour | 8 |
| Premix compounds | 5 |

The premix compounds contain calcium hydrophosphate, salt, choline chloride, phytase, minerals, vitamins, and et al.

Table S5 The basic parameters of eggs from three feeding groups. (one-way ANOVA was performed to evaluate the difference)

| Parameters | CK | FA | AP | P value |
| --- | --- | --- | --- | --- |
| Weight (g) | 47.33±4.02 | 50.20±1.32 | 48.63±2.38 | 0.237 |
| Yolk color | 12.43±1.53^ab^ | 12.55±1.64^a^ | 11.63±1.56^c^ | 0.027 |
| Egg shape index | 1.29±0.04 | 1.30±0.03 | 1.31±0.03 | 0.76 |
| Egg density | 1.12±0.04^c^ | 1.32±0.11^a^ | 1.17±0.08^bc^ | 0.017 |
| Shell strength | 3.58±0.92 | 3.22±0.54 | 3.55±1.06 | 0.067 |
| Thick albumen | 4.67±0.51^b^ | 5.22±0.70^a^ | 4.76±0.50^bc^ | 0.018 |
| Hugh unit | 67.17±5.23 | 73.87±5.37 | 69.12±4.97 | 0.27 |
| Quality | A | AA | A | - |


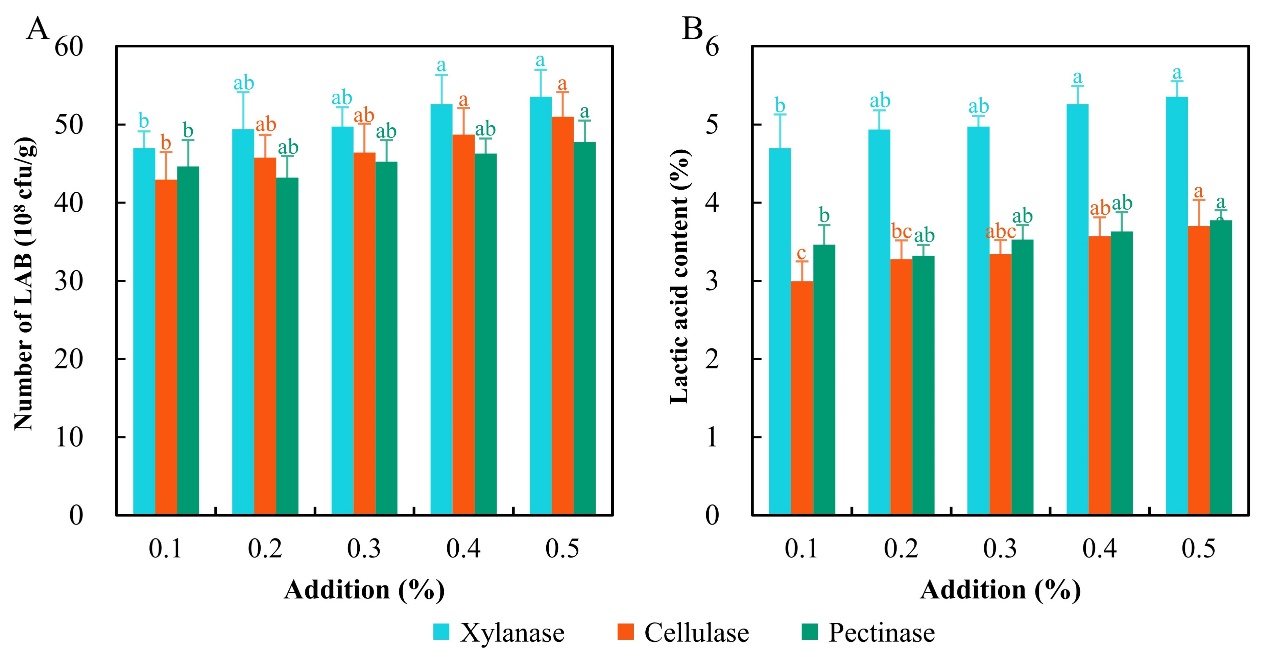


Fig. S1 The optimization of enzyme addition according to the LAB count (A) and lactic acid content (B). (one-way ANOVA was performed to evaluate the difference)
